# H3K9me2 is a determinant for the mitosis-to-meiosis transition in female germ cells

**DOI:** 10.1038/s41419-026-08473-y

**Published:** 2026-03-02

**Authors:** Yanting Hu, Heyang Zhou, Lining Shi, Ke Liu, Xiangyue Meng, Xinru Guo, Liying Shan, Futeng Hu, Yongbin Liu, Teng Zhang, Yang Zhou

**Affiliations:** 1 State Key Laboratory of Reproductive Regulation and Breeding of Grassland Livestock (R2BGL), College of Life Sciences, Inner Mongolia University, Hohhot, 010070 China; 2 Inner Mongolia Agricultural University, Hohhot,, 010018 China

**Keywords:** Pluripotency, Epigenetics

## Abstract

The transition from mitosis to meiosis is crucial for determining the germ cell fate and ensuring the successive production of gametes. However, the mechanisms underlying meiotic entry within the dynamic chromatin context still remain poorly understood. Herein, we demonstrate that H3K9me2, a key marker of heterochromatin formation, plays a pivotal role in the transition from mitosis to meiosis in female germ cells of mice. We show that H3K9me2 maintains high levels in female germ cells from embryonic day 13.5 to 15.5, which closely corresponds to the timing of entry into meiosis in female mice. Interestingly, the reduction of H3K9me2 levels impairs the transition from pluripotency to meiosis in female germ cells, and the role of H3K9me2 appears to act upstream of *Stra8* and *Dazl*. Mechanistically, the multi-omics sequencing analyses of sorted germ cells reveal that H3K9me2 is specifically enriched at the promoter region of pluripotency transcription factor SOX2 and components of the ATP-dependent chromatin remodeling complex. Reduction of H3K9me2 levels results in increased chromatin accessibility, specifically for the pluripotent factor and ATP-dependent chromatin remodelers, thereby impeding the complete exit from the pluripotency progression. Hence, our findings highlight the essential role of H3K9me2 in controlling the exit from the pluripotent state and coordinating the competency of female germ cells, thereby indicating the fundamental role of chromatin remodeling processes in mitosis-to-meiosis transition. This study will provide new insights into the role of chromatin remodeling in the process of gamete production from stem cell to germ cell in vitro.

## Introduction

In mammals, primordial germ cells (PGCs) are the precursors of male and female haploid gametes, which are the only type of cells that can transmit genetic information to the offspring. Meiosis is a core event of sexual reproduction and is responsible for the formation of haploid gametes. The timing of meiotic initiation is different in male and female germ cells. Female germ cells enter meiosis at around E13.5 (embryonic day 13.5), while the meiotic entry of male germ cells occurs after birth in mice [1]. The mitosis-to-meiosis transition activates oogenesis of PGCs, accompanied by downregulation of pluripotency genes, such as *Oct4*, *Sox2*, *Nanog*, and *Dppa3* [1, 2]. During the transition from pluripotency to meiosis, multiple meiosis-specific regulatory factors are coordinately activated and implemented. *Dazl* is an RNA-binding protein expressed in both female and male germ cells and is a key intrinsic factor responsible for meiotic competence [2, 3]. *Dazl*-deficient germ cells fail to enter meiosis normally and remain mitotic state in both female and male mice [2, 3]. *Stra8*, the meiosis gatekeeper gene, is expressed at the preleptotene stage in the embryonic ovary [1, 4]. Retinoic acid (RA) synthesized by the mesonephroi attached to the gonads is an important extrinsic factor in the meiotic initiation through inducing the expression of *Stra8* [1, 4]. However, recent studies have shown some controversial opinions. In RARs-deficient ovaries, *Stra8* and *Sycp3* were expressed in germ cells, and functional oocytes can be produced in the mutant ovaries [5]. Also, the deletion of *Aldh1a*, the ATRA-synthesizing enzyme, does not hinder *Stra8* expression and meiotic initiation [6]. These findings show that RA is dispensable for meiotic initiation, suggesting that there may be other pivotal factors involved in meiotic initiation.

The RNA-binding protein RBM46 is a component of the *Meioc*/*Ythdc2* complex, and testis-specific deletion of RBM46 results in blocked mitosis-to-meiosis transition [7]. Bone morphogenetic protein (BMP), synergistically with RA, primes the competence of meiotic initiation in female germ cells [8]. ZGLP1, a key downstream factor of BMP, is critical for oogenic fate determination, including meiotic initiation, and the ovaries of ZGLP1-deficient female mice were unable to produce oocytes [9]. Recent studies have reported that *Meiosin* interacts with *Stra8* as an upstream regulator, which co-regulates the transition from mitosis to meiosis [10]. *Znhit1* is essential in the meiotic initiation through facilitating the expression of *Meiosin* but not *Stra8* [11]. Thus, meiotic initiation is a complex biological process involving various regulators. However, it is still unclear how meiotic initiation occurs in the context of chromatin.

Before entering meiosis, germ cells undergo mitosis and maintain pluripotency. Hence, the mitotic-to-meiotic transition is a key step in ensuring timely entry into meiotic prophase Ⅰ. Chromatin regulation plays an important role in cell fate determination, correlated with cell type-specific activation or inhibition of gene expression [11, 12]. Chromatin structure influences meiotic recombination [13]. During spermatogenesis and oogenesis, the switch from mitosis to meiosis is accompanied by a highly dynamic chromatin structure and sex-specificity [14–18]. The precise mechanisms through which meiotic initiation is regulated by chromatin remodeling remain largely unknown.

Histone modifications are vital for the proper chromatin structure and gene expression [19–21]. Recent studies have shown that a variety of histone modifications, such as acetylation, methylation, and phosphorylation, are involved in the regulation of meiotic progress [11, 22, 23]. For instance, germline-specific deletion of *Znhit1* in male mice, which is essential for the deposition of histone variant H2A.Z, leads to blockage of meiotic initiation [11]. Histone deacetylation is required for porcine oocyte meiotic resumption by affecting the state of chromatin condensation [24]. γH2AX, formed by phosphorylation of the Ser-139 residue of the histone variant H2AX, is implicated in meiotic DSB repair and is required for chromatin remodeling and inactivation of sex chromosomes in meiotic progression [25, 26].

H3K9me2 is a key marker for heterochromatin formation and gene silencing [27–29]. G9a, a histone methyltransferase, plays a catalytic role in the deposition of H3K9me2 on chromatin during meiotic prophase and is indispensable for synapsis formation in both male and female mice [30]. Additionally, the HP1γ/G9a axis was found to be involved in the synapsis process in male mice [31]. These studies suggest the proposed role for H3K9me2 in the transition from mitosis to meiosis.

To further elucidate the mechanism of chromatin remodeling in the determination of germ cell fate from mitosis to meiosis, we have identified the key histone modification H3K9me2 and established both in vivo and in vitro inhibition systems to demonstrate its functional significance in female mice.

## Results

### H3K9me2 shows high levels in female germ cells after E12.5

**Fig. 1:**
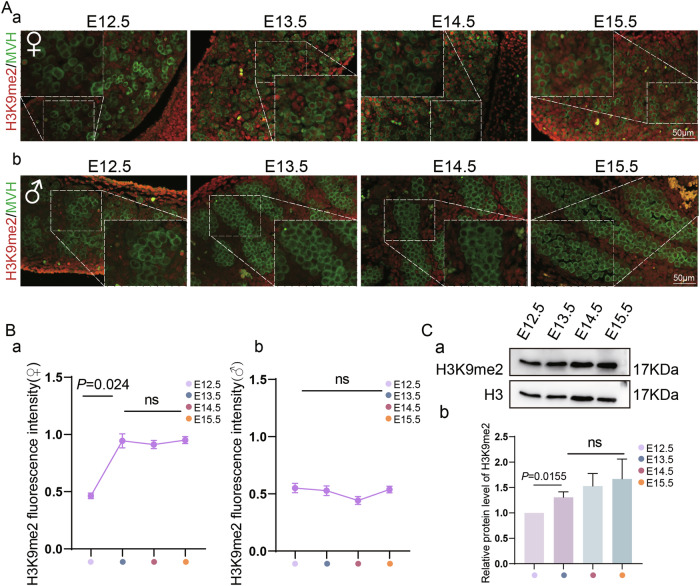
H3K9me2 shows relative high expression in female germ cells after E12.5. **A** Representative sections for immunofluorescence (IF) staining of H3K9me2 in female and male fetal gonads from E12.5 to E15.5. Scale bars = 50 μm. **B** Relative fluorescence intensity of H3K9me2 from E12.5 to E15.5 in both female and male fetal gonads (*n* = 10 biologically independent repeats). **C** (a) Western blot of H3K9me2 in fetal ovaries from E12.5 to E15.5, (b) relative protein levels of H3K9me2 in each fetal stage (*n* = 7 biologically independent repeats).

To investigate the underlying role of histone modification H3K9 in meiotic initiation, we firstly performed H3K9me1/2/3 immunofluorescence staining on fetal gonads from E12.5 (embryonic day 12.5) to E15.5 (Fig. S1A). As shown in Fig. 1, H3K9me2 showed relatively high levels at E13.5 compared to that at E12.5 in the germ cells of female mice (Fig. 1 Aa, Ba); western blot results were consistent with those of immunofluorescence, further confirming that the levels of H3K9me2 were significantly increased after E12.5 in the germ cells of female mice (Fig. 1C), while its levels remained low from E12.5 to E15.5 in the germ cells of male mice (Fig. 1Ab, Bb). In addition, no significant changes were observed in the levels of H3K9me1 and H3K9me3 from E12.5 to E15.5 in both male and female mice (Fig. S1B–D). This implies the elevated levels of H3K9me2 closely correspond to the timing of entry into meiosis in female mice. Given that H3K9me2 is associated with repressive chromatin remodeling, we reasoned that H3K9me2 is involved in the mitotic-to-meiotic transition of female germ cells by downregulating the expression of pluripotency genes.

### H3K9me2 controls the mitosis-to-meiosis transition

**Fig. 2:**
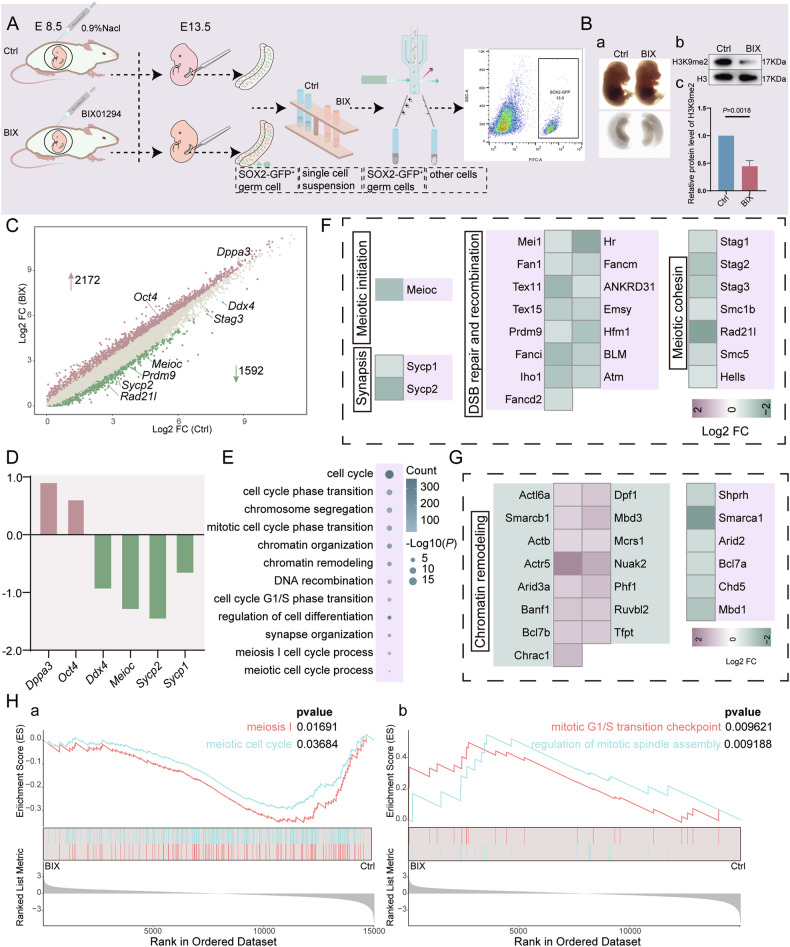
Loss of H3K9me2 leads to defects in the mitosis-to-meiosis transition. **A** Study design diagram of FACS sorting for germ cells. **B** (a) Images of embryos and fetal ovaries at E14.5 in Crtl and BIX. (b, c) Western blot and relative protein levels of H3K9me2 between Crtl and BIX (*n* = 4 biologically independent repeats), H3 is loading control. **C** The scatter plot of the difference genes (log2FC > 0.6) between Ctrl and BIX groups at E13.5. **D** Log2 fold change for pluripotency genes and meiosis related genes. **E** Gene ontology (GO) analysis of difference genes highlighting altered biological processes after H3K9me2 inhibited. **F** Heatmap for downregulated genes of biological processes associated with meiosis according to GO analysis. **G** Heatmap for differential genes of chromatin remodeling. **H** Gene set enrichment analysis (GSEA) associated with meiosis (a) and mitosis (b), with *p*-value < 0.05. Selected gene sets for all genes.

To identify the regulatory mechanisms of H3K9me2 in mitosis-to-meiosis cell cycle transition, we depleted H3K9me2 in vivo by injecting its specific inhibitor BIX01294 at E8.5 and then collected SOX2-positive germ cells from E13.5 female gonads (Fig. 2A, S2A). There was no significant difference in the morphology and size of embryos and gonads between the control (Ctrl) and BIX01294 (BIX) groups at E14.5 (Fig. 2Ba), suggesting that the loss of H3K9me2 does not affect the development of embryos and gonads until E14.5. Western blot analysis further confirmed that H3K9me2 levels were significantly reduced in the BIX group (Fig. 2Bb, c). RNA-sequencing analyses revealed that distinct clustering was apparent between the two groups according to the RNA cluster dendrogram (Fig. S2B). 3764 differentially expressed genes (DEGs) were obtained, of which 2172 genes were upregulated in the BIX group compared with Ctrl group (Fig. 2C). It was shown that the expression of pluripotency genes *Dppa3* and *Oct4* is upregulated, while meiosis-related genes *Meioc*, *Sycp2*, *Sycp1* et al. are downregulated (Fig. 2C, D). Gene ontology (GO) analysis highlighted the DEGs enriched in the terms associated with cell cycle transition, mitotic cell cycle, chromosome segregation, chromatin remodeling and meiotic cell cycle (Fig. 2E). The majority of downregulated genes are involved in meiotic initiation, synapsis, DSB repair and recombination and meiotic condensation (Fig. 2F). Specifically, a total of 21 DEGs related to chromatin remodeling were found, of which 15 genes were upregulated and 6 genes were downregulated (Fig. 2G). Consistent with the defects of mitosis-to-meiosis transition, gene set enrichment analysis (GSEA) indicated that loss of H3K9me2 resulted in a significantly decreased enrichment of genes involved in meiosis I and increased enrichment of genes involved in the mitosis process (Fig. 2H). Taken together, these results suggest that the loss of H3K9me2 caused a blockage of the mitosis-to-meiosis transition.

### H3K9me2 is required for the meiotic progression

**Fig. 3:**
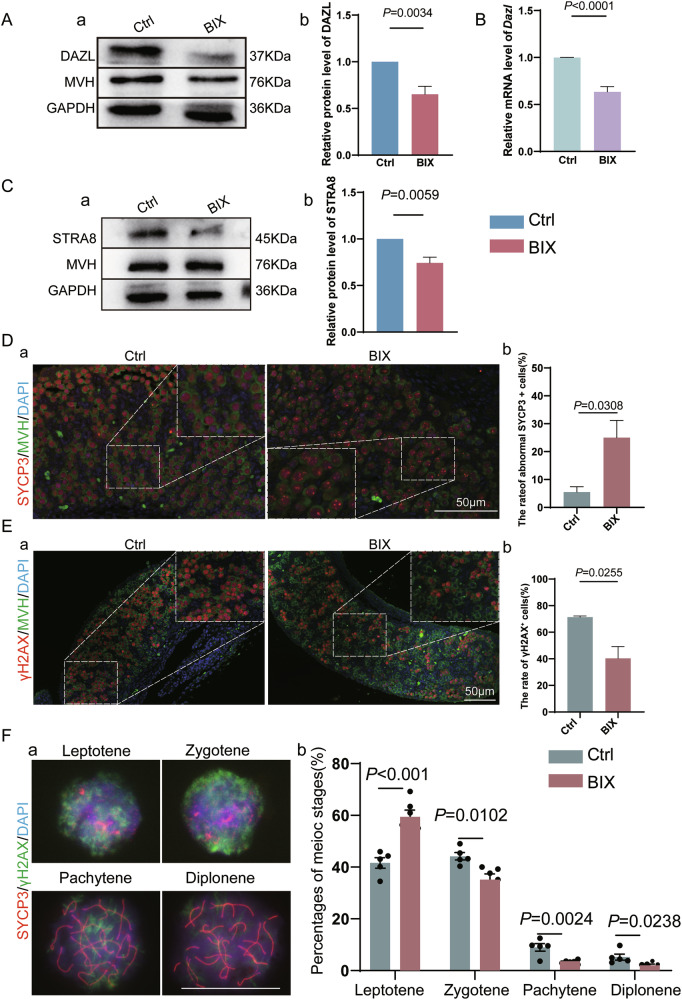
H3K9me2 plays a critical role in meiotic progression. **A** (a) Western blot of DAZL in fetal ovaries between Ctrl and BIX groups at E14.5. (b) Relative protein levels of DAZL from fetal ovaries in Ctrl and BIX groups (*n* = 5 biologically independent repeats), MVH is loading control. **B** RT-qPCR analyses of DAZL in Crtl and BIX at E14.5. **C** (a) Western blot of STRA8 in fetal ovaries between two groups at E14.5. (b) Relative protein levels of STRA8 from fetal ovaries in Ctrl and BIX groups (*n* = 4 biologically independent repeats), MVH is loading control. **D** (a) Representative sections for SYCP3 IF staining from fetal ovaries at E14.5 in Ctrl and BIX groups. Scale bars = 50 μm. (b) The rate of abnormal SYCP3^+^ cells in fetal ovaries (*n* = 4 biologically independent repeats). **E** (a) Representative sections of IF staining for γH2AX in the fetal ovaries from Ctrl and BIX at E14.5. Scale bars = 50 μm. (b) The rate of γH2AX -positive cells in female fetal gonads (*n* = 3 biologically independent repeats). **F** Statistics of staging of meiosis prophase I from E14.5 ovaries between Ctrl and BIX groups. Scale bars = 20 μm.

To define the function of H3K9me2 in meiotic progression, we conducted in vivo injections of mice at E8.5 and dissected the gonads at E14.5. We first examined the expression of genes involved in meiotic initiation, and focused on *Dazl*, a gene essential for STRA8 transcription and germ cell meiotic initiation competence [2]. As shown in Fig. 3A, B, *Dazl* expression was significantly reduced in the BIX group compared to the Ctrl. The expression levels of the meiotic gatekeeper STRA8 were significantly reduced in BIX group compared to Ctrl (Fig. 3C). The immunostaining of SYCP3 (synaptonemal complex protein 3), a component of the synaptonemal complex, showed that a number of SYCP3-positive cells remained, while SYCP3-positive cells predominantly displayed an abnormal punctate pattern in the BIX group compared with the Ctrl group at E14.5 (Fig. 3D). This aberrant pattern persisted until E16.5 (Fig. S3A). At E14.5, immunostaining for γH2AX, a hallmark of DNA double-strand break (DSB) formation, revealed that γH2AX signal was absent in most female germ cells of the BIX group compared to the Ctrl group (Fig. 3E), and this reduction in γH2AX-positive cells persisted at E15.5 (Fig. S3B). To further confirm when meiotic blockage occurred in meiotic prophase I, we performed the meiotic nuclear spreads, and the analysis revealed a significantly increased proportion of the leptotene stage of meiotic prophase Ⅰ in germ cells (Fig. 3F), which suggested developmental defects at the leptotene stage due to loss of H3K9me2. Collectively, these data demonstrated that the meiotic progression of female germ cells was arrested upon H3K9me2 loss.

### Loss of H3K9me2 results in a failure to exit pluripotency process

Female germ cells undergo a dramatic transition from mitosis to entry into the meiotic prophase I at around E13.5 in mice, accompanied by increased expression of *Mvh*, *Stra8*, and *Sycp3* (Fig. S4A). Conversely, the mRNA and protein levels of pluripotency genes *Oct4, Nanog, Sox2*, and *Dppa3* were sharply decreased after E13.5 (Fig. S4B, 4C). These results were consistent with previous studies [3, 32]. As indicated above, the levels of H3K9me2 were increased after E13.5, while the expression of pluripotency genes exhibited the opposite trend. Collectively, the data demonstrated that the levels of H3K9me2 and the expression of pluripotency genes exhibited a coordinated “wax and wane” pattern during the initiation of meiosis.

**Fig. 4:**
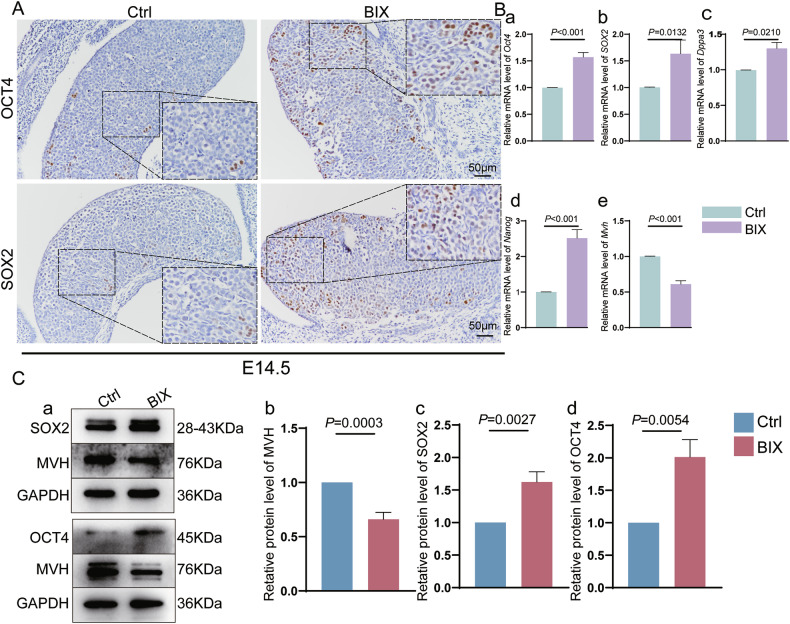
Loss of H3K9me2 leads to the inability to exit the pluripotency state in vivo. **A** Representative sections for immunohistochemistry (IHC) staining of OCT4 and SOX2 at E14.5 between Ctrl and BIX groups. Scale bars = 50 μm. **B** (a–e) RT-qPCR analyses of *Oct4* (*n* = 6 biologically independent repeats), *Sox2* (*n* = 3 biologically independent repeats), *Dppa3 (n* = *6* biologically independent repeats*)*, *Nanog* (*n* = 3 biologically independent repeats) and *Mvh* (*n* = 12 biologically independent repeats) genes at E14.5 in Ctrl and BIX. MVH and GAPDH are loading controls. **C** (a) Western blot of SOX2, OCT4, MVH from Ctrl and BIX. (b–d) Relative protein levels of MVH (*n* = 6 biologically independent repeats), SOX2 (*n* = 6 biologically independent repeats) and OCT4 (*n* = 5 biologically independent repeats), GAPDH and MVH are loading controls.

Next, we sought to define the pluripotency gene expression profile of female germ cells following H3K9me2 loss in vivo. Immunostaining revealed that SOX2- and OCT4- positive germ cells were markedly increased in the BIX group compared with the Ctrl group (Fig. 4A). RT-qPCR and western blot results were consistent with the immunostaining findings (Fig. 4Ba–d, 4Ca, c, d), which further confirmed a dramatic increase in the expression of pluripotency genes, as opposed to the levels of MVH in the BIX group as shown in Fig. 4Be, Cb, implying a loss of germ cells due to depletion of H3K9me2.

To ensure that BIX01294 acts specifically on the gonad, we cultured female gonads in vitro. The Ctrl group was cultured in a basal medium while the BIX group was supplemented with an additional 5uM H3K9me2 inhibitor, BIX01294. The results of immunohistochemistry, RT-qPCR and western blot showed that the pluripotency genes *Oct4*, *Sox2*, *Dppa3* and *Nanog* were retained high expression at E14.5 (Fig. S5A–C).

The above results were consistent with the phenotype that pluripotency gene expression was not reduced in *Dazl*-deficient (*Dazl*^−/−^) and *Stra8*-deficient (S*tra8*^−/−^) mice at E13.5 and E15.5 (Fig. S6A, B). Furthermore, to explore the relationship between H3K9me2 and DAZL (a key intrinsic factor for meiosis) as well as STRA8 (the gatekeeper of meiosis), fetal ovaries from *Dazl*^−/−^ and S*tra8*^−/−^ mice were dissociated into cells and immunofluorescence staining for H3K9me2 was performed at E14.5. Analysis of relative fluorescence intensity revealed that H3K9me2 levels in germ cells from *Dazl*^−/−^ and S*tra8*^−/−^ mice did not show significant differences compared to wild-type mice (Fig. S7), indicating that H3K9me2 was potentially upstream of *Stra8 and Dazl*.

These results indicated that loss of H3K9me2 led to the majority of germ cells failing to enter meiosis and maintaining their pluripotent state.

### Loss of H3K9me2 leads to aberrant exit from the mitotic cell cycle

**Fig. 5:**
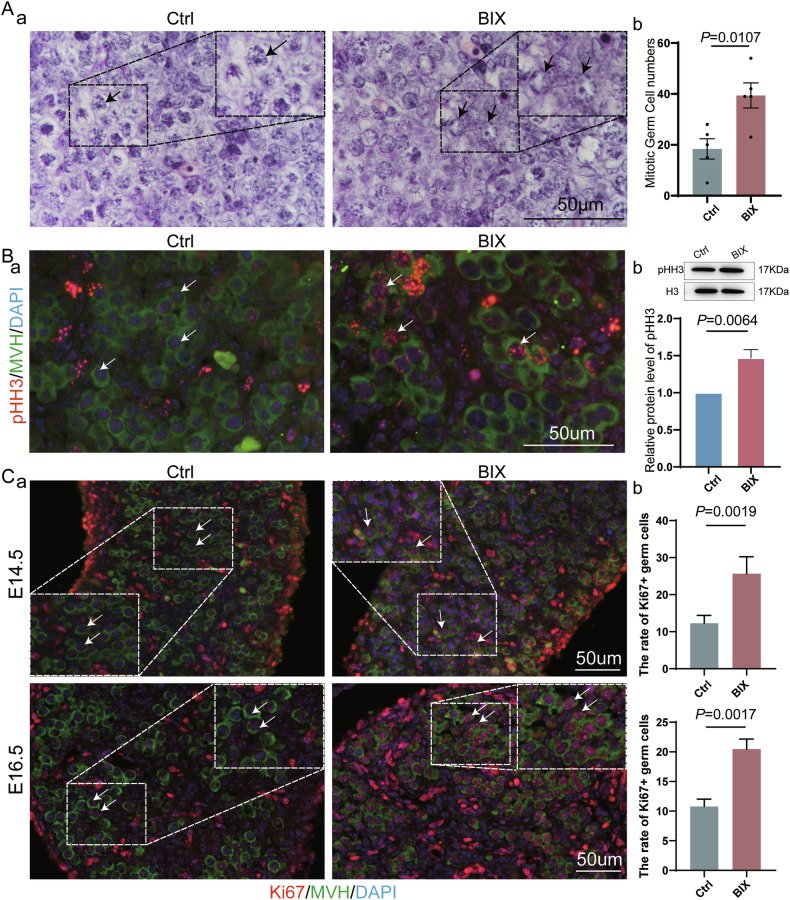
H3K9me2 is essential for the normal exit from the mitotic cell cycle program. **A** (a) H&E staining of representative sections within fetal ovaries from E14.5 between Ctrl and BIX. (b) The number of mitotic cells in Ctrl and BIX (*n* = 5 biologically independent repeats). **B** (a, b) Representative image of IF staining, western blot and relative protein levels of for pHH3 at E14.5 female fetal gonads from Ctrl and BIX groups (*n* = 4 biologically independent repeats). **C** (a) Image is representative for IF staining of Ki67 between Crtl and BIX groups at E 14.5 and E16.5. All images scale bars = 50 μm. (b) The rate of Ki67^+^ germ cells in E14.5 (*n* = 4 biologically independent repeats) and E16.5 (*n* = 5 biologically independent repeats) fetal ovaries between Ctrl and BIX groups.

To delineate the fate of germ cells that failed to enter meiosis upon the loss of H3K9me2, we further compared nuclear morphology in the Ctrl and BIX groups at E14.5. The majority of germ cells undergo chromosome condensation in Ctrl, a characteristic of meiosis prophase Ⅰ. By contrast, the proportion of germ cells in preleptotene that did not display such condensation in BIX was significantly higher than that in the Ctrl (Fig. 5A).

Phosphorylated histone H3 on serine-10 (pHH3) is a marker for cell proliferation and is specific to mitosis [33]. The weak expression of pHH3 was observed in only a few germ cells from the Ctrl fetal ovaries. In comparison, the expression of pHH3 was relatively strong in BIX at E14.5, as demonstrated by immunofluorescence staining and western blot analysis (Fig. 5Ba, b). Subsequently, we examined Ki67, a nuclear protein expressed in cycling cells. Through immunofluorescence co-staining of Ki67 with MVH, we found that germ cells in Ctrl ovaries showed weakly positive expression of Ki67, whereas the majority of germ cells in BIX ovaries were Ki67 positive at E14.5 and E16.5 (Fig. 5C), indicating that these germ cells remained in the mitotic cell cycle. Collectively, the loss of H3K9me2 led to an incorrect exit from the mitotic cell cycle in germ cells.

TUNEL staining was performed on the fetal ovaries of both groups to investigate the fate of germ cells that failed to enter meiosis. The number of TUNEL-positive cells was increased in the BIX gonads compared with the Ctrl group at E14.5 and E16.5 (Fig. S8A). Consistent with this elevated apoptosis, we observed a significant reduction in both the number of MVH-positive germ cells and MVH protein levels (Fig. S8B, C). These findings indicate that germ cells that failed to enter meiosis properly were undergoing apoptosis.

### H3K9me2 directly regulates genes associated with pluripotency and chromatin remodeling

**Fig. 6:**
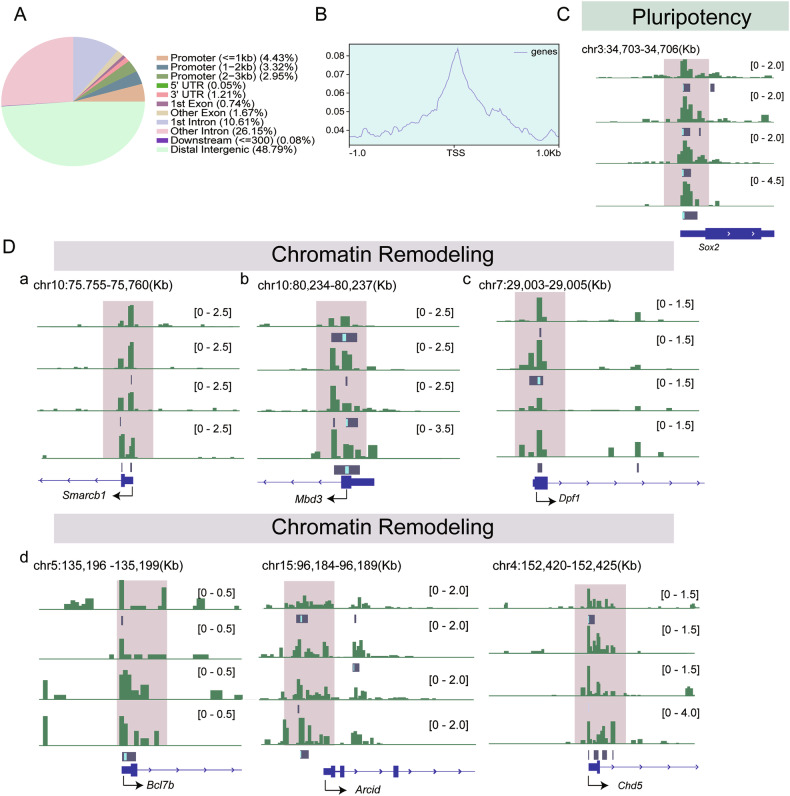
H3K9me2 directly occupies the promoter region of genes associated with pluripotency and chromatin remodeling. **A** Distribution of H3K9me2 binding sites in the genome in wild-type female germ cells. **B** CUT&RUN-sequencing signals spanning the entire genome visualized in a TSS-centric manner. **C** CUT&RUN- sequencing normalized reads shown for pluripotency genes *Sox2*. **D** CUT&RUN- sequencing normalized reads shown for chromatin remodeling genes *Smarcbl*, *Mbd3*, *Dpf1*, *Bcl7b*, *Arcid*, and *Chd5*.

To investigate the mechanisms by which H3K9me2 drives the mitosis to meiosis transition, we analyzed CUT&RUN sequencing data for H3K9me2 from published data of female germ cells in wild-type mice at E14.5 [34]. Our analysis revealed that 10.7% of genes corresponding to H3K9me2-enriched regions were located within promoter regions (Fig. 6A, B). To determine whether the DEGs from our RNA-sequencing data were direct H3K9me2 targets, we analyzed these DEGs using CUT&RUN data. Interestingly, it was shown that H3K9me2 was directly enriched at the promoter region of the pluripotency gene *Sox2* (Fig. 6C), as well as numerous subunits of ATP-dependent chromatin remodelers (e.g., *Smarcb1*, *Mbd3*, *Dpf1*, *Bcl7b*, *Arid*, and *Chd5*) (Fig. 6D), all of which showed significant differences between the two groups in the RNA-sequencing analysis. However, it is noteworthy that the meiotic genes, such as *Stra8*, *Sycp3* and *Dazl*, were not identified as direct targets of H3K9me2 (Fig. S9).

These analyses suggest that the regulation of mitosis-to-meiosis transition by H3K9me2 involves occupying the promoter regions and silencing the key pluripotency transcription factor SOX2 and subunits of ATP-dependent chromatin remodeling complex, rather than being directly enriched at meiotic genes.

### H3K9me2 modulates the mitosis-to-meiosis transition by altering chromatin accessibility

**Fig. 7:**
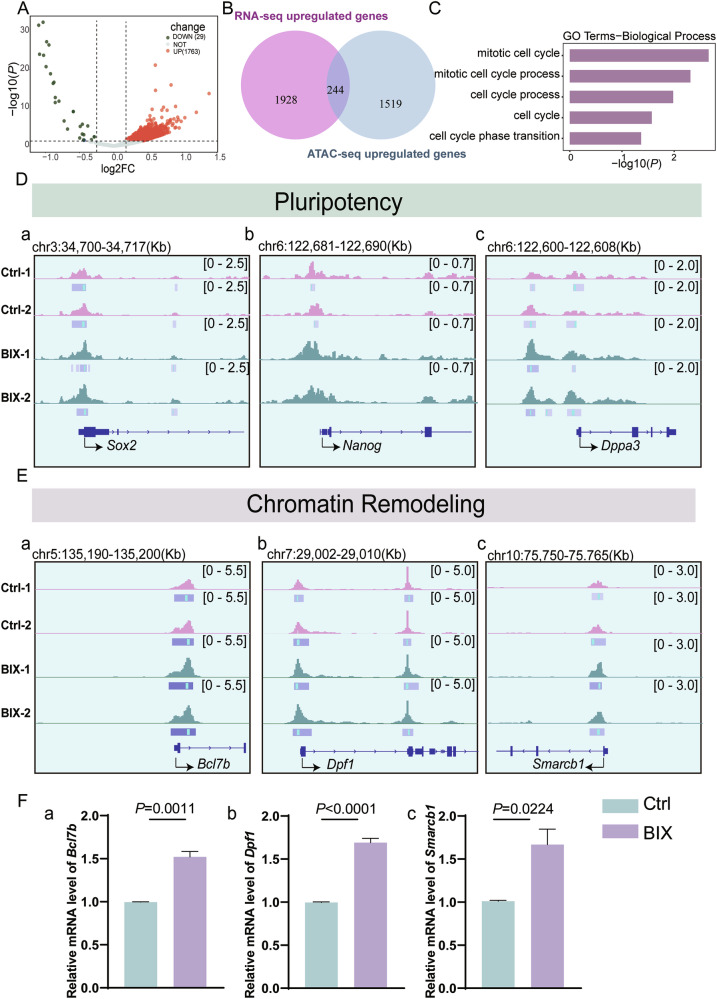
H3K9me2 alters chromatin accessibility to regulate the transition from mitosis to meiosis. **A** Volcano plot of the genes associated with differential accessibility regions (DARs) between Ctrl and BIX groups (*n* = 2 biologically independent repeats). **B** Venn diagram of overlapping upregulated genes between RNA-sequencing and ATAC-sequencing. **C** GO enrichment analysis of overlapping upregulated genes from RNA-sequencing and ATAC-sequencing. **D** ATAC-sequencing normalized reads shown for pluripotency genes *Sox2*, *Nanog* and *Dppa3*. **E** ATAC-sequencing normalized reads shown for chromatin remodeling genes *Bcl7b*, *Dpf1* and *Smarcb1.* F (a–c) RT-qPCR analyses of *Bcl7b* (*n* = 3 biologically independent repeats), *Dpf1* (*n* = 3 biologically independent repeats), *Smarcb1* (*n* = 3 biologically independent repeats) genes at E14.5 in Ctrl and BIX.

Chromatin remodeling refers to the molecular mechanism that alters the packaging state of chromatin, including histones within nucleosomes and corresponding DNA molecules. This process regulates gene expression by modulating the chromatin accessibility. Remodeling factor complexes play crucial roles in nucleosome remodeling [35]. Recent studies have confirmed the essential role of ATP-dependent chromatin remodelers in maintaining pluripotency in embryonic stem cells (ESCs) [36–46]. CUT&RUN-sequencing analysis has revealed that H3K9me2 is enriched at the genomic loci of multiple ATP-dependent chromatin remodeling factors, underscoring the necessity for examining chromatin accessibility under H3K9me2 modulation. To observe changes in chromatin accessibility following H3K9me2 loss, ATAC-sequencing was conducted, using the same method as that for isolating germ cells for RNA-sequencing. A total of 1792 genes associated with differentially accessible regions (DARs) were identified between Ctrl and BIX groups, of which 1763 were significantly upregulated and 29 were significantly downregulated (Fig. 7A). This observation aligns with the known biological function of H3K9me2 in suppressing gene expression. Given the predominance of increased chromatin accessibility in the ATAC-sequencing data, our integrated analysis focused on genes with upregulation in RNA sequencing and linked to enhanced chromatin accessibility in ATAC sequencing. This approach identified 244 overlapping genes (Fig. 7B), and GO enrichment analysis demonstrated significant enrichment in cell cycle-related processes, including “mitotic cell cycle”, “cell cycle”, and “cell cycle phase transition”, which is consistent with our experimental validation (Fig. 7C). Building on our previous combined analyses of RNA-sequencing and CUT&RUN-sequencing, we further evaluated changes in ATAC-sequencing peaks of pluripotency- and chromatin remodeling-related genes, specifically, the peaks of *Sox2*, *Dppa3*, *Nanog*, *Bcl7b*, *Dpf1*, and *Smarcb1* in the BIX group were higher than those in the Ctrl group (Fig. 7D, E). In addition, RT-qPCR results showed that the mRNA levels of *Bcl7b*, *Dpf1*, and *Smarcb1* were significantly increased in the BIX group (Fig. 7F).

Loss of H3K9me2 leads to increased chromatin accessibility, specifically for genes such as *Sox2* and chromatin remodelers *Bcl7b*, *Dpf1*, and *Smarcb1*. This increase is consistent with the findings from RNA-sequencing and CUT&RUN-sequencing analysis, indicating that these genes are modified by H3K9me2 at their promoter regions. These results underscore the significance of H3K9me2 in the mitosis-to-meiosis transition in female germ cells, potentially through direct regulation by pluripotent transcription factors and modulation by ATP-dependent chromatin remodelers.

## Discussion

The mitosis-to-meiosis transition is a complex process of cell fate regulation, accompanied by extensive chromatin remodeling; however, the role of chromatin dynamics in germ cells remains poorly understood. In our study, by using in vivo and in vitro inhibition systems in conjunction with molecular and multi-omics sequencing analyses, we present molecular and functional evidence that H3K9me2 serves as a crucial regulator of the mitosis-to-meiosis transition. Our findings provide insights into how specific chromatin dynamics influence cell fate determination.

*Stra8*, the gatekeeper gene of meiotic initiation, exhibited significant changes following the loss of H3K9me2 in our study. Additionally, no significant differences in H3K9me2 levels were observed in female germ cells from S*tra8*^−/−^ mice compared to wild-type mice. These results indicate that H3K9me2 regulates the mitosis-to-meiosis transition by acting upstream of *Stra8*, suggesting a regulatory role for H3K9me2 in meiotic initiation. Furthermore, the loss of H3K9me2 resulted in reduced expression of *Dazl*, a key intrinsic factor for meiotic competence. Interestingly, the levels of H3K9me2 did not show a significant change in germ cells from *Dazl*^−/−^ ovaries. This implies that H3K9me2 acts as an upstream regulator of *Stra8* and *Dazl*, potentially playing a pivotal role in regulating the meiotic process.

Before meiotic initiation, primordial germ cells (PGCs) are in a mitotic state and express pluripotency genes. Switching off the expression of pluripotency genes is a pivotal aspect of the mitosis-to-meiosis transition. In the present study, we found that loss of H3K9me2 resulted in the blockage of the meiotic progression and a failure to fully repress the expression of pluripotency genes. Through a combined analysis of RNA-sequencing, CUT&RUN-sequencing and ATAC-sequencing, we identified that H3K9me2 is enriched at various chromatin remodeler subunits, which are essential for the establishment and maintenance of embryonic stem cell (ESC) pluripotency.

PGCs exhibit developmental pluripotency, while embryonic stem cells (ESCs) possess totipotency. These distinct cell types show unique characteristics alongside shared features, including the expression of key pluripotency factors such as *Sox2*, *Oct4* and *Nanog* [47]. The mechanisms underlying ESC pluripotency and self-renewal have attracted considerable research interest. DNA methylation, histone modification, and ATP-dependent chromatin remodeling represent the primary chromatin regulatory pathways known to govern the status of ESCs [35, 48]. Various chromatin remodeling complex components have been identified to play crucial roles in regulating the state of ESCs [36–42]. ATP-dependent chromatin remodeling complexes can be categorized into four families based on the structural features of the ATPase catalytic subunit: SWI/SNF, ISWI, CHD, and INO80 complexes [35].

In mammals, the SWI/SNF complex is also known as BAF [38]. Specifically, within embryonic stem cells (ESCs), the esBAF complex contains subunits such as BRGl, SRG3, ARID1A, ARID1B, SMARCB1, and ACTL6a. Studies have shown that depletion of these subunits affects the expression of pluripotency genes [36–46]. The esBAF complex interacts with gene promoters regulated by key pluripotency factors *Oct4, Sox2*, and *Nanog*, playing a crucial role in establishing and maintaining ESC pluripotency [37, 38, 44–46]. It is plausible to suggest that ATP-dependent chromatin remodelers play a similar role in primordial germ cells (PGCs). Furthermore, it has been demonstrated that the loss of the core subunits of the BAF complex, BRG1, and INO80, results in the halting of meiosis I. Furthermore, the CHD and ISWI complexes are essential for the post-meiosis processes [49–52]. However, limited research exists on how chromatin remodeling factors regulate pluripotency in PGCs. In this study, we identified that H3K9me2 is enriched at several subunits of chromatin remodeling enzymes via CUT&RUN-sequencing analysis, coinciding with the sustained expression of pluripotency genes in E14.5 female germ cells. These findings indicate that H3K9me2 may regulate germ cell pluripotency by targeting multiple subunits of chromatin remodeling complexes. Loss of H3K9me2 resulted in upregulation of *Actl6a* and *Smarcb1* in the RNA-sequencing data. Additionally, ATAC-sequencing results showed an increased peak for *Smarcb1*. These genes have been reported to be critical for the function of key pluripotent transcription factors. Furthermore, significant changes were detected in other chromatin remodeling subunits in the RNA-sequencing results for the BIX group. The CUT&RUN-sequencing analysis also revealed that H3K9me2 is enriched at multiple ATP-dependent chromatin remodeling factors. Although little research has been conducted on these factors, they potentially play a key role in the transition from mitosis to meiosis. A recent study by Zhao et al. demonstrated that H3K9me2 eliminates ST18-mediated transcriptional repression of meiotic genes [53]. and we show its parallel role in suppressing the pluripotent state. These complementary roles highlight the regulatory functions of H3K9me2 in coordinating female germ cell development. While this study offers insights into the role of H3K9me2 in regulating pluripotency in germ cells, the precise mechanisms involved require further exploration.

## Materials and methods

### Animal breeding and model

Animals used were ICR mice. The mice were fed a standard diet and kept under a 12/12 h light/dark cycle with 22 ± 2 °C, 45–60% humidity according to the national guideline. The mating time was scheduled at 4:30–5:30 p.m., and the vaginal plug was checked at 8:30–9:30 a.m. The next day, using 8-week-old female mice, the presence of vaginal plugs was defined as E0.5 (embryonic day 0.5). Pregnant mice were randomized to receive normal saline (Ctrl group) or BIX01294 (50mg/kg) (BIX group) by a one-time intraperitoneal injection at E8.5. *Dazl*^−/−^ and *Stra8*^−/−^ murine models, along with SOX2-GFP mice, are on a C57BL/6 background.

### Organ culture

Before formal culture, the agarose gel pieces (1.5% w/v) must be preincubated with DMEM/F12 for at least 24 h in a 24-well plate. The E12.5 gonads of female fetal mice were separated, then placed on agarose stands in 24-well plates. They were then cultured in DMEM containing 10% FBS in an incubator at 37 °C and 5% CO_2,_ and changed half of the culture medium every 24 h, while an additional 5 μM H3K9me2 inhibitor, BIX01294, was added to the culture medium in the BIX group. After 72 h, the cultured gonads were collected for subsequent assay.

### Morphology and immunofluorescence/immunohistochemistry

E14.5 fetal ovaries were fixed overnight in Bouin’s solution (for hematoxylin and eosin staining) or 4% formaldehyde (for immunohistochemistry), dehydrated by passing through gradient ethanol, embedded in paraffin, and sectioned at 5 μm thickness of slices. For morphological analysis, the sections were stained with hematoxylin and eosin.

For immunofluorescence, the sections were placed in sodium citrate boiling for 10 min. Samples were then blocked for 45 min with 5% donkey serum and incubated with primary antibody diluted in 5% donkey serum overnight at 4 °C. The slides were washed three times with PBS buffer and incubated with secondary antibodies labeled with a fluorescent group at room temperature for 1 h. Nuclei were treated with DAPI at room temperature for 5–10 min. Finally, the slides were mounted with fluorescence anti-quencher. All images were taken using a Nikon fluorescence microscope (A1, Japan).

For immunohistochemistry, before secondary antibody incubation, the sections were treated with 3% hydrogen peroxide for 10 minutes. The blocking solution was 5% BSA, and after the secondary antibody incubation was completed, the samples were treated with DAB chromogenic solution. Counterstaining was treated with hematoxylin 5 min. The primary antibodies used are as follows: MVH (abcam, AB13840), H3K9me1 (abcam, ab9045), H3K9me2 (ABclonal, A2359), H3K9me3 (abcam, ab8898), SYCP3 (abcam, ab97672), γH2AX (ABclonal, AP0099), STRA8 (abcam, ab49602), OCT4 (abcam, ab181557), DPPA3 (abcam, ab19878), SOX2 (Proteintech, 11064-1-AP), Ki67 (abcam, ab15580), pHH3 (Millipore, 06-570).

### Relative fluorescence intensity analysis

Nikon fluorescence microscope (A1, Japan) was employed for image acquisition of stained sections under strictly controlled optical parameters to ensure inter-section comparability. For relative quantification, germ cell and adjacent somatic cell regions were demarcated using ImageJ with manual threshold adjustment. The relative fluorescence intensity was calculated by germ cell /somatic cell.

### Chromosome spread and staining

Collected fetal ovaries of E14.5 were immersed in HEB for 2 h. Next, the gonads were shredded with tweezers to release the germs cells in 0.1 M sucrose solution droplet. The samples were fixed overnight in 1% paraformaldehyde. Fixed samples were blocked in antibody dilution buffer (ADB) for 30 min at 37 °C and incubated with primary antibody for 6-8 h. After three times washes with TBS, slides were blocked by ADB overnight at 4 °C. The next day, the slides incubated with secondary antibody for 2.5 h at 37 °C. Nuclei were stained with DAPI for 5-10 min, then washed three times. Slides were sealed with fluorescence anti-quencher. At least 300 germ cells were counted to analyze the different stages of chromosomes during meiosis prophase I and imaging on Nikon fluorescence microscope.

### TUNEL assay

TUNEL assay was performed using a DeadEnd^TM^ Fluorescence TUNEL System (Promega, G3250). According to instructions, store in a humidified environment at 37 °C for 60 min with sufficient rTdT incubation buffer away from direct light. Cover the tissue with a coverslip to ensure even distribution of reagents. Cell nuclei were counterstained with DAPI.

### Western blot

Fetal ovaries were collected and proteins were extracted in RIPA buffer supplemented with protease inhibitors on ice. After denaturation, the protein extracts were electrophoresed on SDS-PAGE gels, and subsequently transferred to a PVDF membrane. After blocking with 5% skim milk powder for 1 h at room temperature, the primary antibody was incubated overnight at 4 °C. Finished three times of cleaning with 1×TBST buffer, the secondary antibody was incubated at room temperature for about 1 h, washed three times with TBST, then exposed using a BeyoECL Plus Kit (Beyotime, A0018). The primary antibodies used are as follows: MVH (abcam, AB13840, 1:1000), H3 (Proteintech, 17168-1-AP, 1:1000), DAZL (abcam, ab34139), others are consistent with antibodies used in immunostaining.

### RT-qPCR

RNA was extracted from fetal ovaries using RNeasy micro columns (QIAGEN, 74004) and cDNA generated using a reverse transcription kit (TaKaRa, RR047A). RT-qPCR was performed using TB Green Premix Ex Taq (TaKaRa, RR820A) in 10ul reaction system. The primer for *Mvh* (forward: 5’-AGGGGATGAAAGAACTATGGTC-3’, and reverse: 5’-AGCAACAAGAACTGGGCACT-3’), *Gapdh* (forward: 5’-GTCATTGAGAGCAATGCCAG-3’, and reverse: 5’- GTGTTGCTACCCCCAATGTG-3’), *Dppa3* (forward: 5’-GACCCAATGAAGGACCCTGAA-3’,and reverse: 5’- GCTTGACACCGGGGTTTAG-3’), *Nanog* (forward: 5’-AGGCTTTGGAGACAGTGAGGTG-3’, and reverse 5’-TGGGTAAGGGTGTTCAAGCACT-3’), *Sox2* (forward: 5’-CATCCACTTCTACCCCACCTT-3’, and reverse: 5’-AGCTCCCTGTCAGGTCCTT-3’), *Oct4* (forward: 5’-AGAGGATCACCTTGGGGTACA-3’, and reverse: 5’-CGAAGCGACAGATGGTGGTC-3’).

### Germ cells acquisition and isolation

Fetal ovaries were isolated from pregnant female mice at E13.5, then placed in PBS on ice. The gonads were dissociated with Trypsin EDTA solution (Sigma-Aldrich, T4049) at 37 °C for 3-5 min, terminated with 10% FBS diluted in DMEM/F12. After being filtered by a cell strainer, centrifuged at low speed in a 4 °C centrifuge, and resuspended in DPBS, germ cells and somatic cells were separated by FACS based on expression of SOX2-GFP. The germ cells isolated can be continued for further assay or frozen at -80 °C.

### RNA library creation and sequencing

Germ cells were separated by FACS based on expression of SOX2-GFP at E13.5 female gonads, then approximately 8,000 cells per duplicate were collected. Total RNA underwent oligo-dT/SMART-mediated reverse transcription. Synthesized double-stranded cDNA was purified, quality-controlled (Agilent 2100), and fragmented for library construction. Sequencing using the DNBSEQ platform for 2 biological duplicates, to obtain paired end 100 bp reads (PE100).

After trimming to remove low-quality ends and adapter sequence using Trim Galore (version 0.6.7), the resulting reads were mapped to the mouse genome using Hisat2 (version 2.2.1). Differential expression analysis was performed using DESeq2 (version 1.36.0) in R. Genes with an absolute value of log2 fold changes greater than 0.6 and a *p*-value < 0.05 are considered significant differential genes. GO enrichment analysis were performed using g:Profiler website (https://biit.cs.ut.ee/gprofiler). GSEA was performed using clusterProfiler (version 4.4.4).

### ATAC library creating and sequencing

Germ cells were obtained as described above at E13.5, and approximately 10,000 cells were collected per biological duplicate. Next, prepare the ATAC library according to the instructions of the Hyperactive ATAC-Seq Library Prep Kit (Vazyme, TD711) and the TruePrep Index Kit (Vazyme, TD202). We used Illumina NovaSeq for sequencing, with paired-end reads of 150 bp (PE150).

The trimmed ATAC- sequencing reads were aligned to reference genomes using BWA (version 0.7.17-r1188) with default parameters. Converting the generated SAM file to BAM format SAMtools (version 1.15.1) was used. Peak calling was performed using MACS2 (version 2.2.7.1). Differentially accessible regions (DARs) were obtained by using package DESeq2 (version 1.36.0) in R, with p-value < 0.05. GO enrichment analysis were performed using g:Profiler website (https://biit.cs.ut.ee/gprofiler). The genome browser IGV (http://software.broadinstitute.org/software/igv/) was used to examine the ATAC-sequencing tracks.

### CUT&RUN-sequencing data analysis

The ChIPseeker (version 1.32.0) R packages were used to obtain distance to the nearest transcriptional start site (TSS). The others are consistent with ATAC-sequencing.

### Statistics analysis

Each experiment in this study was conducted at least three times. GraphPad Prism was used for statistical analysis. Biological assay results were expressed as mean ± SEM. Student’s unpaired t-test was used to determine the significant difference between two groups and multiple comparison tests were determined by one-way analysis of variance (ANOVA). All the results were statistically significant with *p*-value < 0.05.

The sample size was determined based on similar studies in this field. Investigators were not blinded to allocation during the experiments, use of animals or outcome assessment. No samples were excluded from analysis unless specified.

## Supplementary information

Supplementary Figures legends

Western Blot

Figure S1

Figure S2

Figure S3

Figure S4

Figure S5

Figure S6

Figure S7

Figure S8

Figure S9

## Data Availability

RNA-seq and ATAC data have been deposited in the Genome Sequence Archive (GSA, https://ngdccncb.ac.cn/gsa) under the accession number CRA021062 and CRA021102. All data are available in the main text or the supplementary materials.

## Code availability

All the code is available in our GitHub repository (https://github.com/Zhouyang106/H3K9me2-data-analysis.git).

## Data availability

All data are available in the main text or the supplementary materials.
