# Supplementary material for: H3K9me2 is a determinant for the mitosis-to-meiosis transition in female germ cells
Source: Cell Death Dis. 2026 Mar 2;17(1):289. doi: 10.1038/s41419-026-08473-y (PMC13031797; doi:10.1038/s41419-026-08473-y)
Supplement: Supplementary file 1 — Supplementary Figures legends [file 41419_2026_8473_MOESM1_ESM.docx]

**Supplementary Figures legends**

**Fig. S1. H3K9me1/3 show no significant alteration from E12.5 to E15.5 in both female and male mice. A** The images for bright field of wild type fetal gonads from E12.5 to E15.5 in both female and male. **B** IF staining of representative sections for H3K9me1 from E12.5 to E15.5 in fetal gonads of both female and male. **C** Representative images of IF staining for H3K9me3 from E12.5 to E15.5 in fetal gonads of both both female and male. **D** (a-d) Relative fluorescence intensity of H3K9me1/3 from E12.5 to E15.5 in both female and male fetal gonads (n=10 biologically independent repeats). All scale bars = 50 μm.

**Fig. S2. SOX2-GFP positive germ cells and RNA cluster dendrogram. A** (a) The images of bright field and FITC channel for fetal gonads in both female and male mice at E13.5. Scale bars = 50 μm. (b) The images of sorted female germ cells for SOX2^+^. **B** The RNA cluster dendrogram of Crtl and BIX groups (n=2 biologically independent repeats).

**Fig. S3. H3K9me2 is critical for the progression of meiosis. A** (a) Representative images for SYCP3 IF staining at E15.5 and E16.5 from fetal ovaries between Ctrl and BIX group. Scale bars = 50 μm. (b) The rate of abnormal SYCP3^+^ cells in fetal ovaries at E15.5 (n=6 biologically independent repeats) and E16.5 (n=3 biologically independent repeats). **E** (a) Representative sections of IF staining for γH2AX in the fetal ovaries between Ctrl and BIX group at E15.5. Scale bars = 50 μm. (b) The number of γH2AX^+^ cells in female fetal ovaries at E15.5 (n=3 biologically independent repeats).

**Fig. S4. The expression of meiosis and pluripotency genes from E12.5 to E15.5 in wild-type ovaries. A** (a-c) RT-qPCR analyses of *Mvh*, *Stra8* and *Sycp3* genes from E12.5 to E15.5 wild-type ovaries (n=3 biologically independent repeats), MVH is loading control. **B** (a-d) RT-qPCR analyses of *Oct4*, *Sox2, Dppa3* and *Nanog* genes from E12.5 to E15.5 wild-type ovaries (n=3 biologically independent repeats), MVH is loading control. **C** (a) Western blot of OCT4, SOX2 and DPPA3 from E12.5 to E15.5 wild-type ovaries. (b-d) Relative protein levels of OCT4 (n=4 biologically independent repeats), SOX2 (n=4 biologically independent repeats) and DPPA3 (n=5 biologically independent repeats), MVH is loading control.

**Fig. S5. Loss of H3K9me2 leads a failure to exit pluripotency process *in vitro*. A** Representative images of IHC staining for OCT4, SOX2 and DPPA3 at E14.5 between Ctrl and BIX. Scale bars = 50 μm. **B** (a-d) RT-qPCR analyses of *Oct4*, *Sox2*, *Dppa3* and *Nanog* genes at E14.5 in Ctrl and BIX (n=3 biologically independent repeats), MVH is loading control. **C** (a-f) Western blot and relative protein levels of H3K9me2 (n=5 biologically independent repeats), OCT4 (n=3 biologically independent repeats) and SOX2 (n=3 biologically independent repeats) in Ctrl and BIX, H3 and MVH are loading control.

**Fig. S6. The expression of pluripotency genes remains high in *Dazl* ^-/-^ and *Stra8* ^-/-^mice.** **A** Representative IHC staining images for OCT4, SOX2 and DPPA3 of E13.5 and E15.5 fetal ovaries in *Dazl* ^+/+^ and *Dazl* ^-/-^ mice. **B** Representative sections of IHC staining for OCT4 and SOX2 from E13.5 and E15.5 fetal ovaries in *Stra8* ^+/+^ and *Stra8* ^-/-^ mice. All scale bars = 50 μm.

**Fig. S7. H3K9me2 shows no significant changes in *Dazl*** **^-/-^ and *Stra8* ^-/-^ mice.** Representative IF staining images of dissociated single cells for H3K9me2 at E14.5 from wild type, *Dazl* ^-/-^ and *Stra8* ^-/-^ fetal ovaries (n=100 independent cells). Scale bars = 50 μm.

**Fig. S8. Germ cells not entering meiosis properly are undergoing apoptosis.** A (a) TUNEL staining of representative sections within fetal ovaries between the Ctrl and BIX groups at E14.5 and E16.5. Scale bars = 50 μm. (b) The numbers of TUNEL^+^ germ cells numbers in E14.5 (n=3 biologically independent repeats) and E16.5 (n=3 biologically independent repeats) fetal ovaries between Ctrl and BIX groups. B IF staining of MVH in representative fetal ovarian sections from E16.5 mice between the Ctrl and BIX groups. Scale bars = 50 μm. C Western blot and relative protein levels of MVH (n=5 biologically independent repeats) in Ctrl and BIX. MVH is loading control.

**Fig. S9**. **Meiotic genes are not direct targets of H3K9me2**. (a-c) CUT&RUN-sequencing normalized reads shown for meiotic genes *Stra8*, *sycp3* and *Dazl*.
