# Supplementary figures and images for: H3K9me2 is a determinant for the mitosis-to-meiosis transition in female germ cells
Source: Cell Death Dis. 2026 Mar 2;17(1):289. doi: 10.1038/s41419-026-08473-y (PMC13031797; doi:10.1038/s41419-026-08473-y)

Western Blot

Fig. 1C

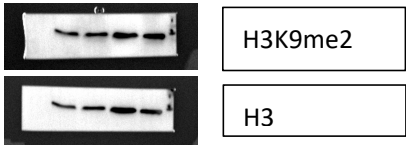

Fig. 2Bb

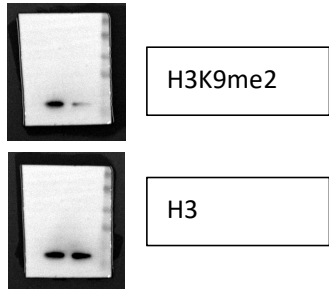

Fig. 3Aa

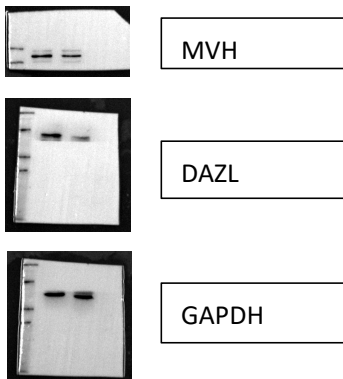

Fig. 3Ca

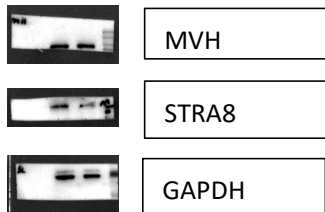

Fig. 4Ca

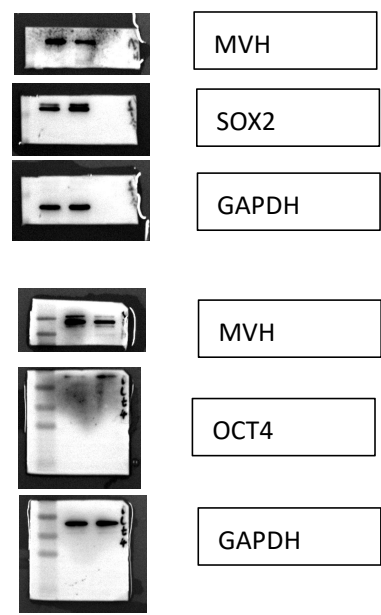

Fig. 5Bb

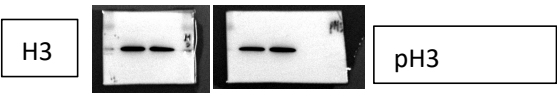

Fig. S4Ca

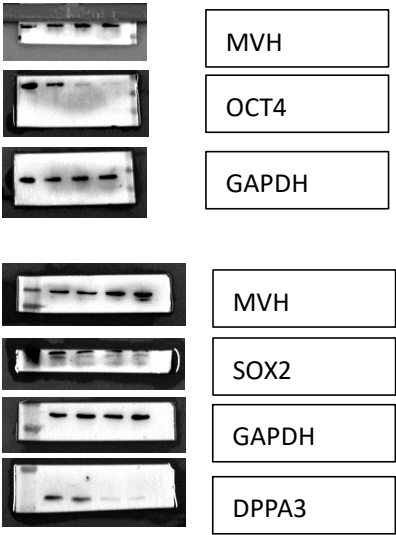

Fig. S5C

a

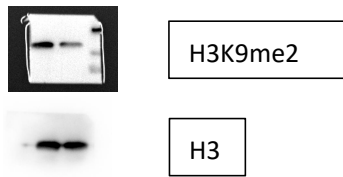

b

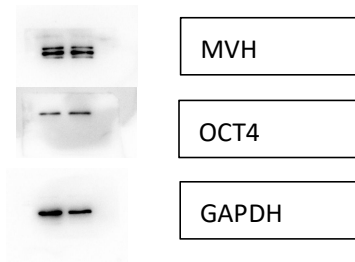

c

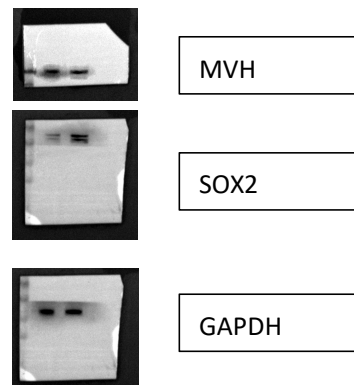

Fig. S8C

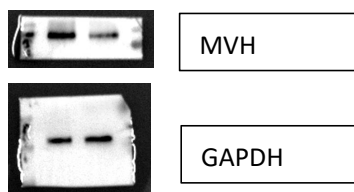

Supplement: Supplementary file 2 — Western Blot [file 41419_2026_8473_MOESM2_ESM.pdf]

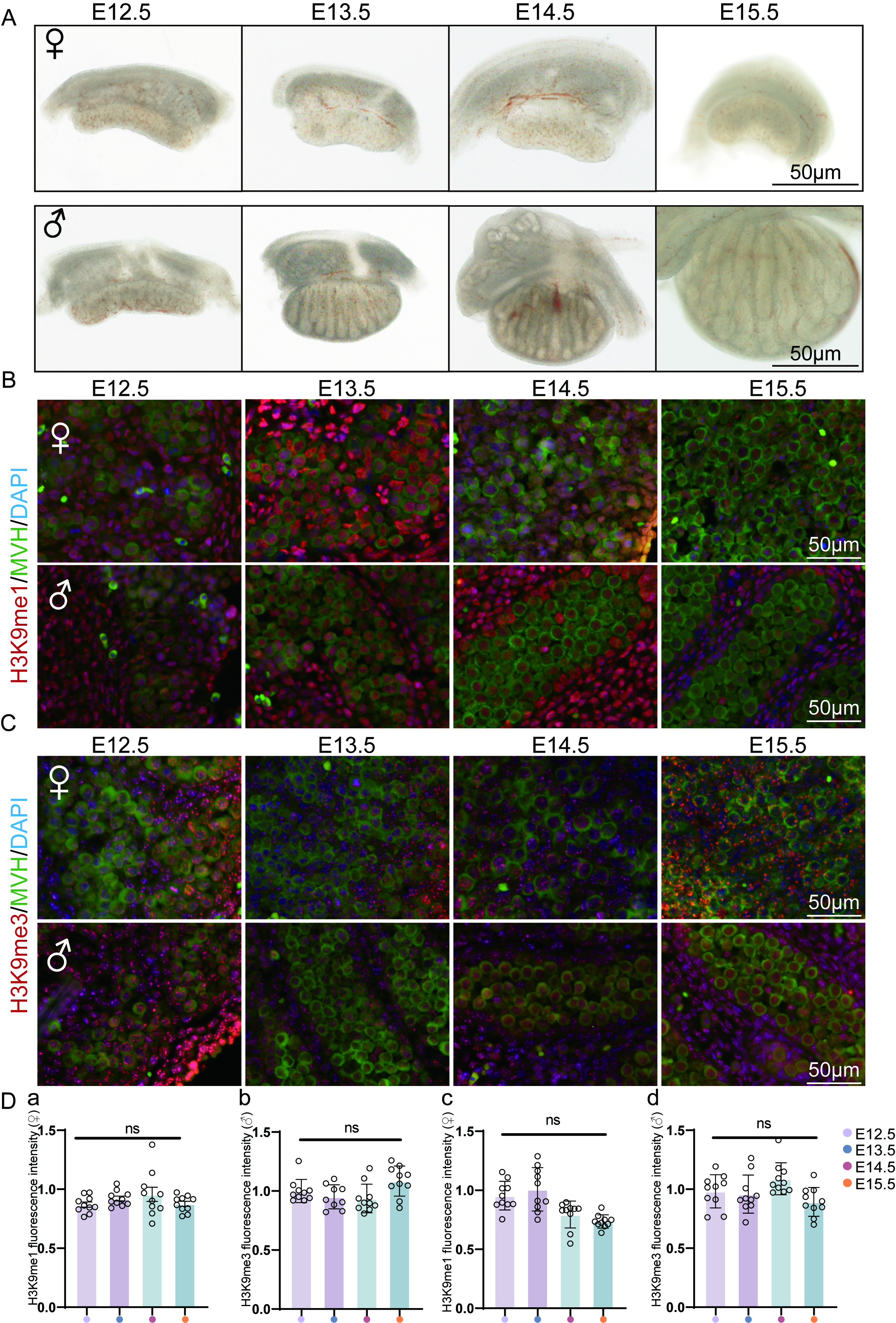

Supplement: Supplementary file 3 — Figure S1 [file 41419_2026_8473_MOESM3_ESM.tif]

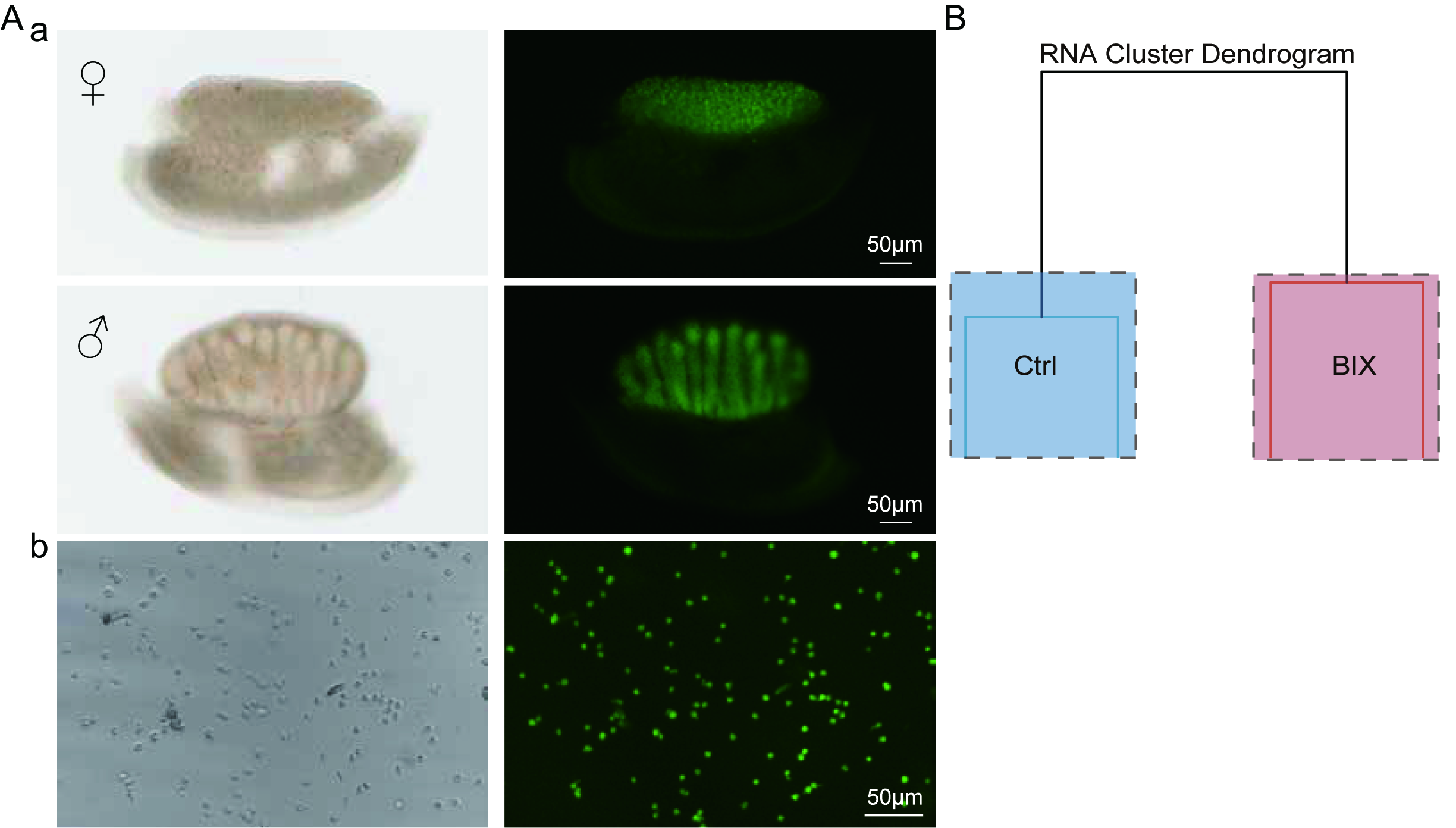

Supplement: Supplementary file 4 — Figure S2 [file 41419_2026_8473_MOESM4_ESM.tif]

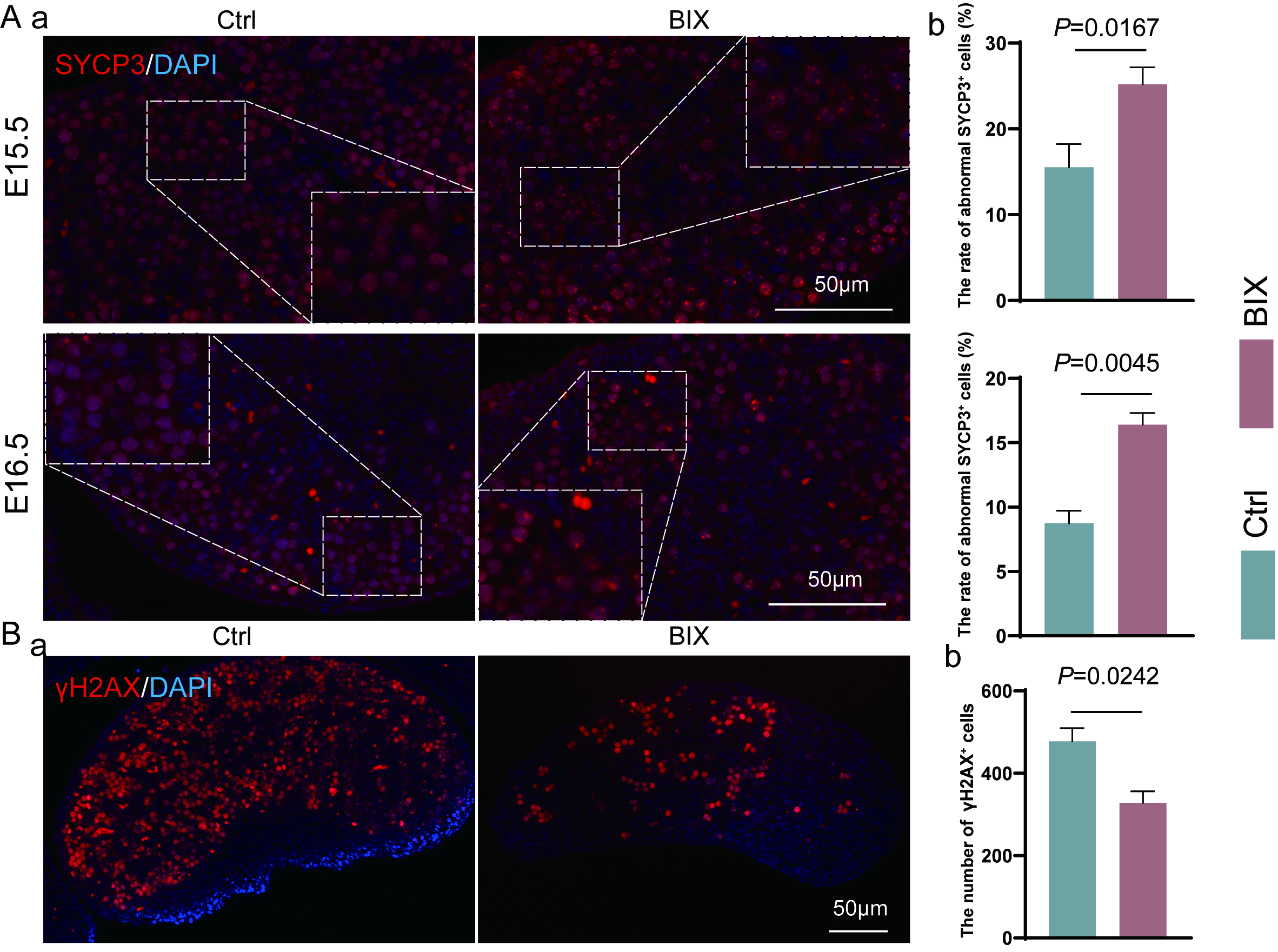

Supplement: Supplementary file 5 — Figure S3 [file 41419_2026_8473_MOESM5_ESM.tif]

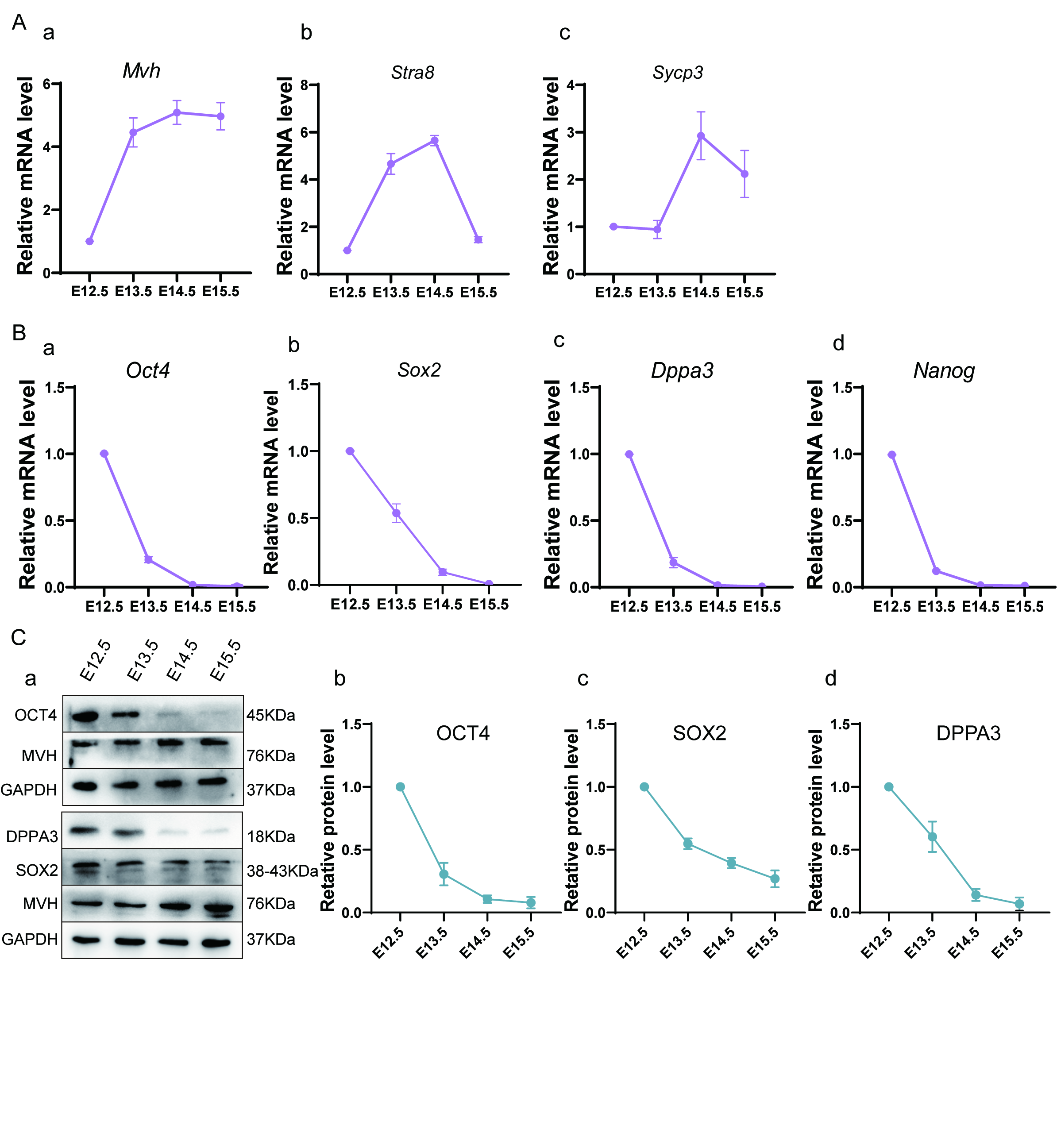

Supplement: Supplementary file 6 — Figure S4 [file 41419_2026_8473_MOESM6_ESM.tif]

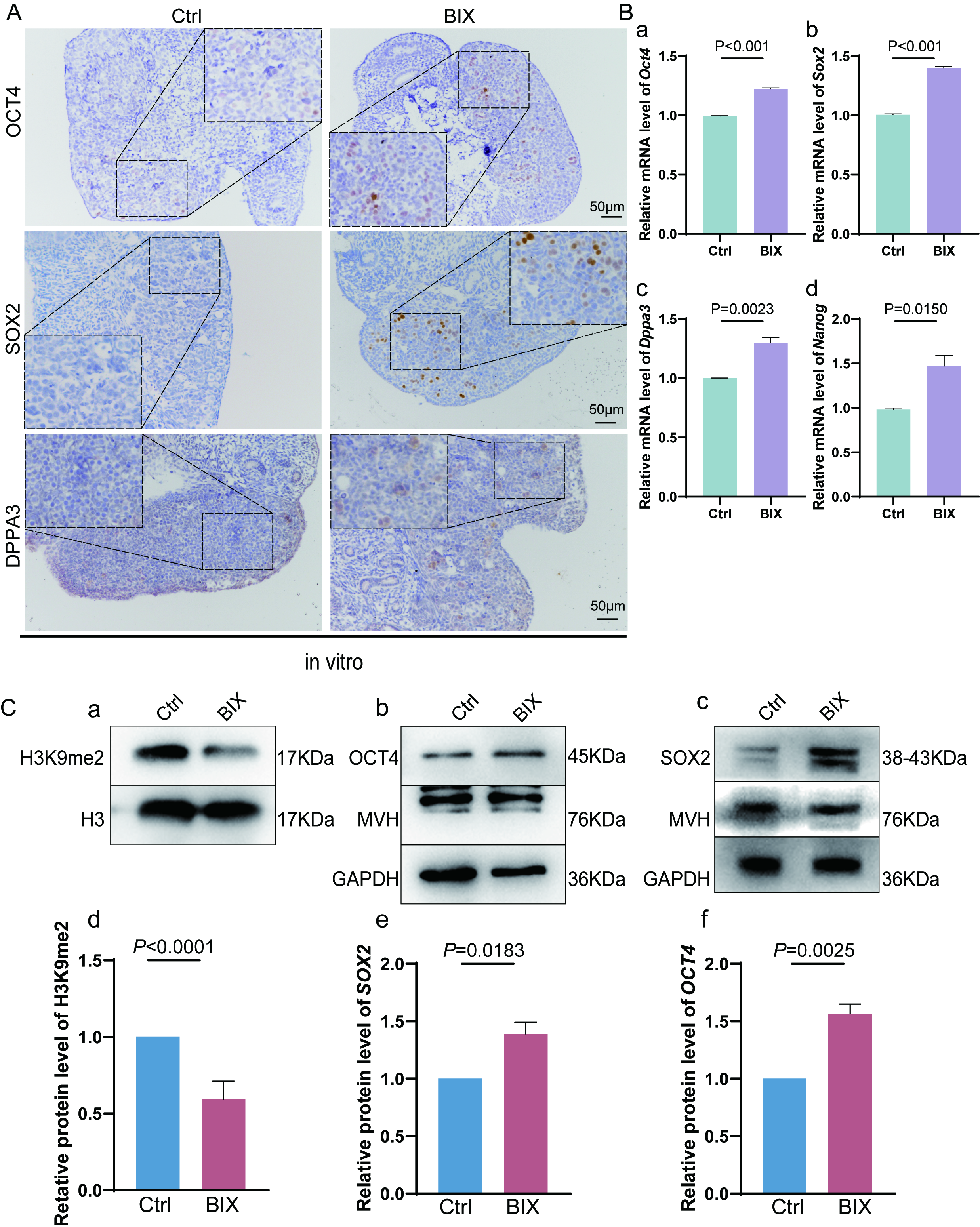

Supplement: Supplementary file 7 — Figure S5 [file 41419_2026_8473_MOESM7_ESM.tif]

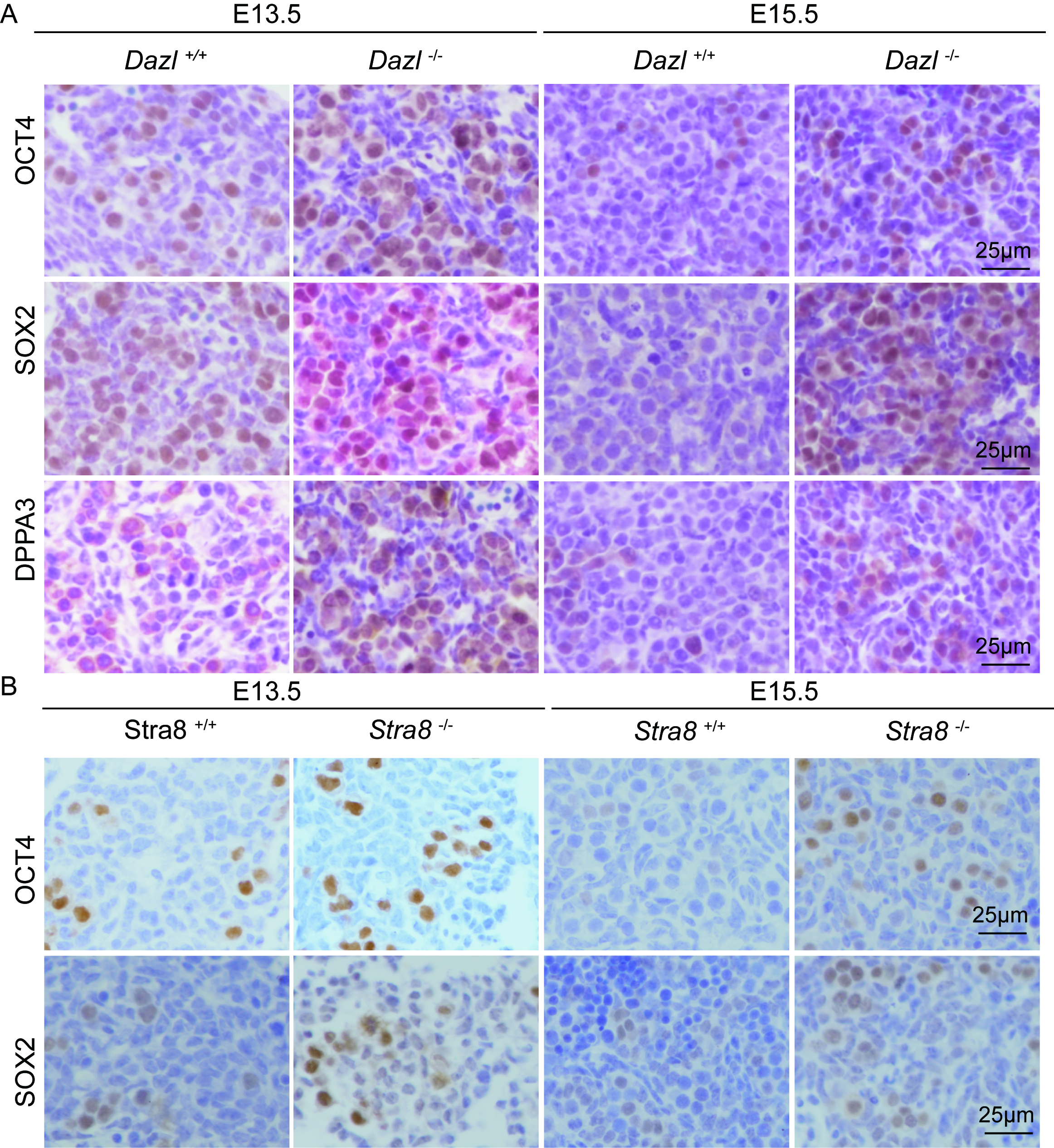

Supplement: Supplementary file 8 — Figure S6 [file 41419_2026_8473_MOESM8_ESM.tif]

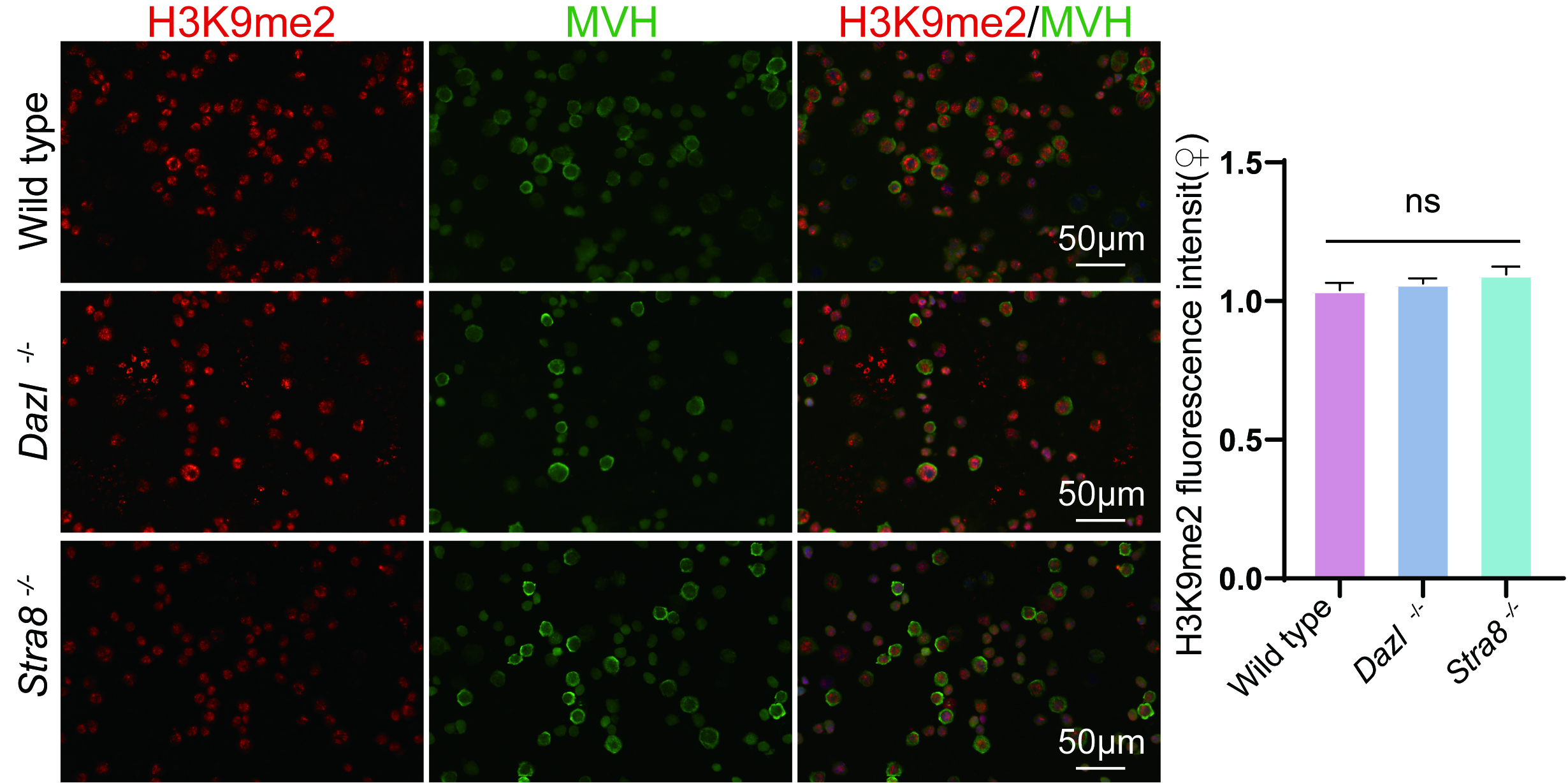

Supplement: Supplementary file 9 — Figure S7 [file 41419_2026_8473_MOESM9_ESM.tif]

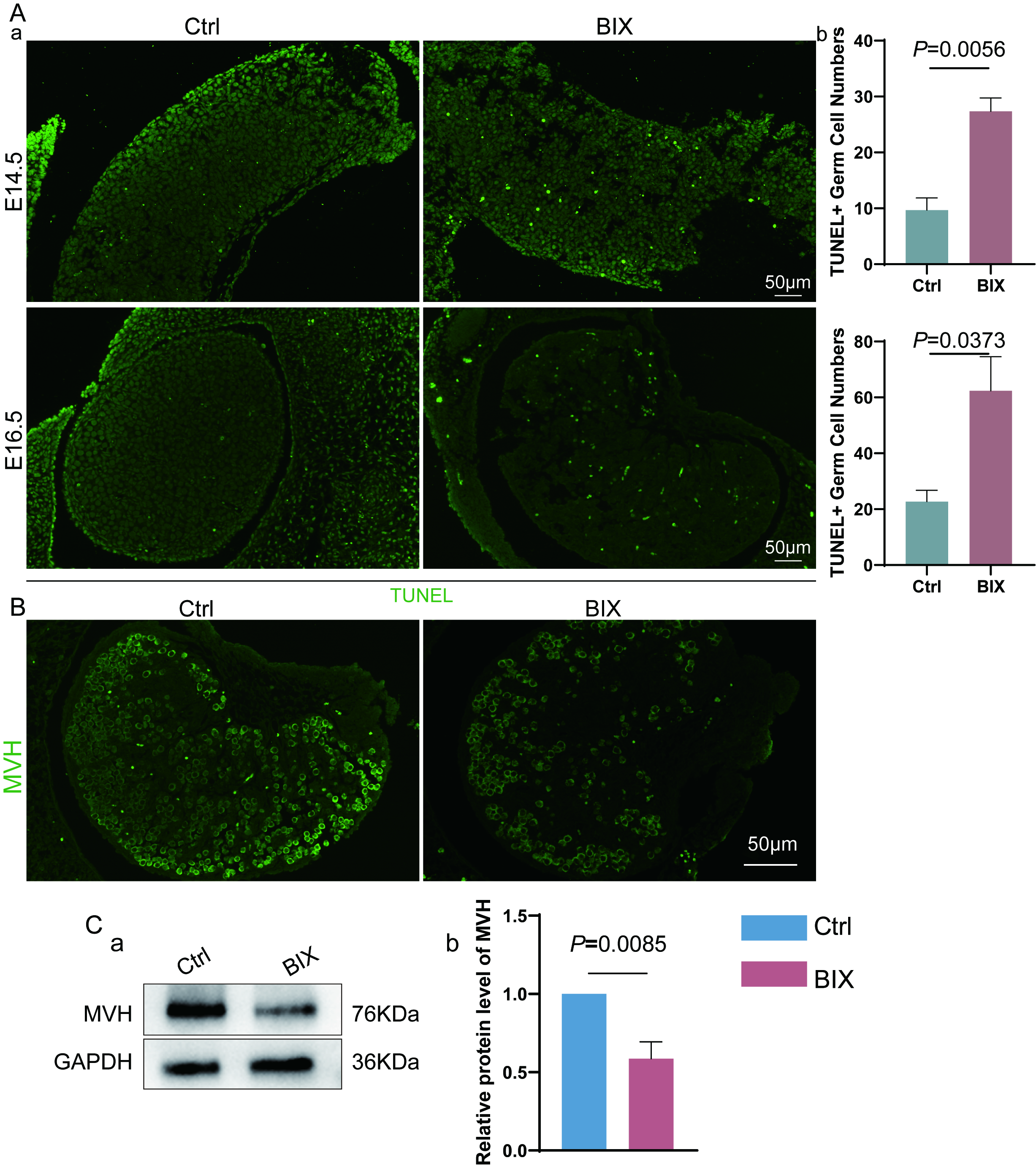

Supplement: Supplementary file 10 — Figure S8 [file 41419_2026_8473_MOESM10_ESM.tif]

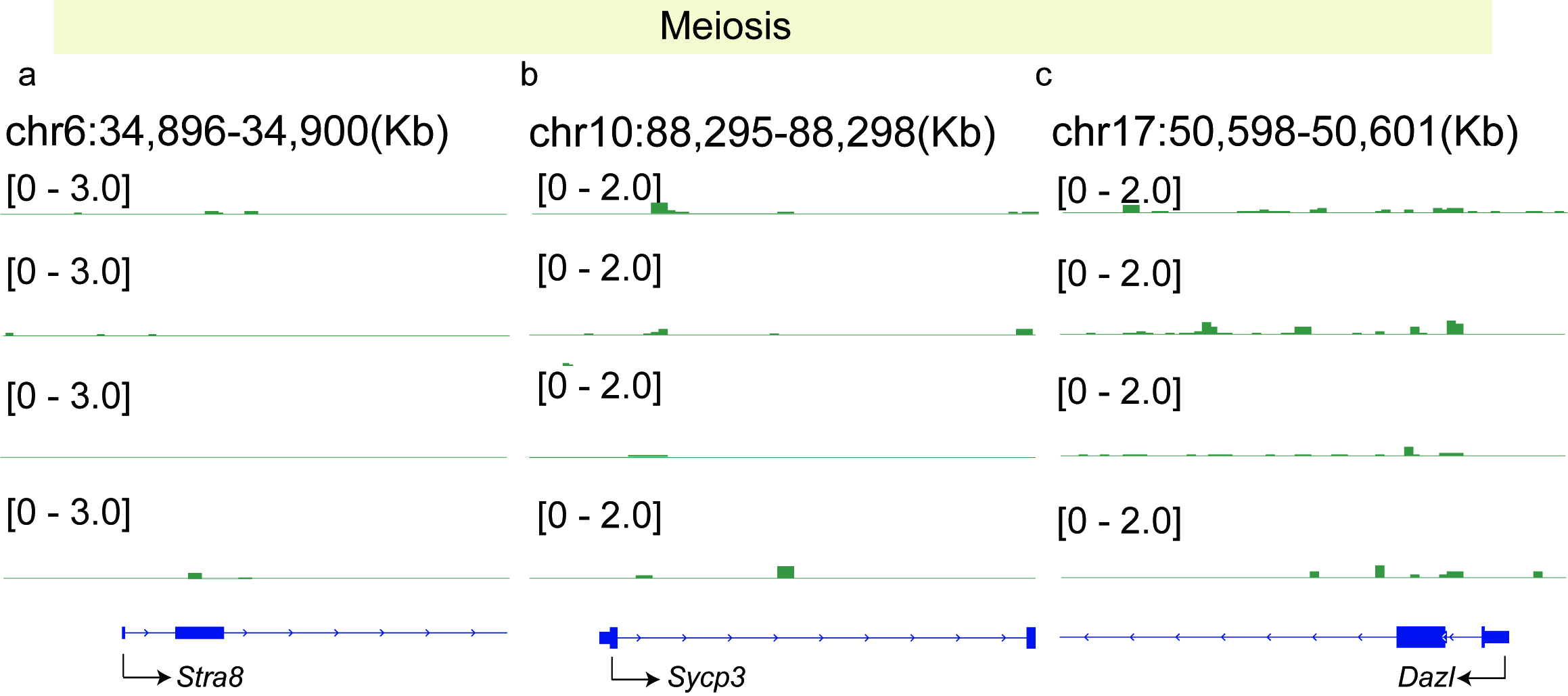

Supplement: Supplementary file 11 — Figure S9 [file 41419_2026_8473_MOESM11_ESM.tif]
